# Progressive evolution of secondary aquatic adaptation in hippos and cetaceans

**DOI:** 10.1038/s41421-022-00483-2

**Published:** 2022-12-20

**Authors:** Lei Chen, Zihe Li, Baosheng Wu, Botong Zhou, Rasmus Heller, Jiong Zhou, Kun Wang, Zeshan Lin, Dongdong Wu, Qiang Qiu

**Affiliations:** 1 School of Ecology and Environment, Northwestern Polytechnical University, Xi’an, Shaanxi China; 2 Center for Evolutionary Hologenomics, GLOBE Institute, University of Copenhagen, Copenhagen, Denmark; 3 State Key Laboratory of Genetic Resources and Evolution, Kunming Institute of Zoology, Chinese Academy of Sciences, Kunming, Yunnan China

**Keywords:** Comparative genomics, Molecular biology

Dear Editor,

Long after the common ancestor of all terrestrial vertebrates crawled to land from the sea during the Late Devonian, a small fraction of their descendants returned to aquatic habitats, which has been labeled “secondary aquatic adaptation”. How these species regained the ability to thrive in aquatic habitats has been of great interest to evolutionary biologists. The fully-aquatic cetaceans and semi-aquatic hippos, collectively forming the clade Whippomorpha, provide an excellent comparative model for investigating the secondary aquatic adaptation^1^. One crucial question remains for this taxon: whether their common ancestor was semi-aquatic. The most parsimonious evolutionary hypothesis is that the common ancestor of Whippomorpha was already semi-aquatic, and this is supported by several shared aquatic characters, especially the hairless skin. Alternatively, cetaceans and hippos recolonized aquatic environments independently by evolving from a terrestrial common ancestor^2^. Of note, a recent study found that inactivating mutations in skin-related genes occurred independently in hippos and cetaceans^2^, and another study found only weak evidence for positive selection in the common ancestor of Whippomorpha^3^. However, the inactivation of genes is only one of many routes to phenotypic evolution, and genome assembly quality can significantly impede the power to detect selection in ancestral branches. Hence, the sequence of evolutionary events during this prominent case of secondary aquatic adaptation remains unresolved.

Comparative genomics is an effective way for determining the molecular mechanisms of adaptive traits^4^. To examine this fabled instance of secondary aquatic adaptation, we generated high-quality genome assemblies for the only two extant hippo species, the common hippo (*Hippopotamus amphibius*) and the pygmy hippo (*Choeropsis liberiensiss*). The assembled genomes of common hippo and pygmy hippo are 2423 Mb and 2374 Mb, with contig N50 of 73.3 Mb and 20.2 Mb, respectively (Supplementary Fig. S1 and Tables S1,S2). About 95% of the common hippo contigs were anchored to 18 chromosomes (Supplementary Fig. S2) using Hi-C data, and a pseudo-chromosome was assembled for pygmy hippo using the common hippo genome as the reference (Supplementary Fig. S3). The synteny map and high coverage (95.3% and 94.7%) of complete BUSCO genes indicate high contiguity and integrity (Fig. 1a). Multiple chromosome fusion events could explain the lower haploid chromosome number in the common hippo (Fig. 1a). Repetitive sequences and protein coding genes were then identified (Supplementary Table S3). Compared to the published common hippo genome (GCA_004027065.1, contig N50 of 74,418 bp), we filled 28,329 of 31,130 N-gaps and added 130.8 Mb of novel sequences (5% of the genome). Notably, 5949 of the filled gaps map within genes (Supplementary Fig. S4). The phylogenetic trees based on 11,274 orthologous genes and whole-genome alignments of 9 mammalian species confirm sister relationships of hippos and cetaceans, which diverged at ~53.2 Ma (55.3 to 47.9 Ma; Supplementary Fig. S5). We found that the effective population of hippos declined from around 1 Ma (Supplementary Fig. S6), consistent with the general aridification of Africa, which might have reduced the aquatic habitat to hippos.

**Fig. 1:**
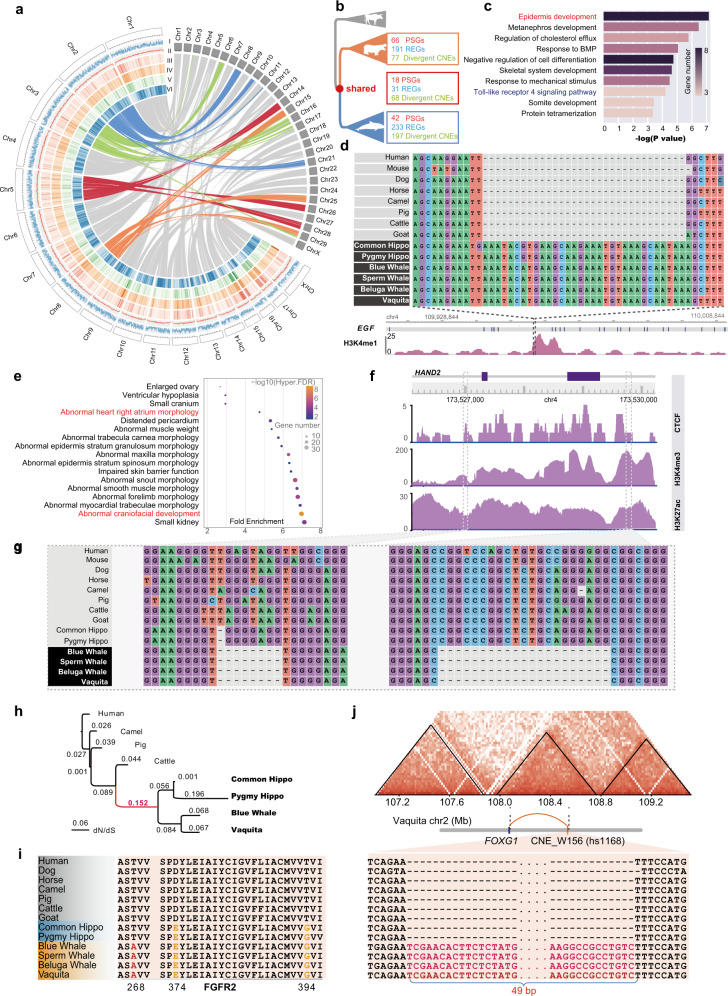
Adaptive evolution of the Whippomorpha. **a** Syntenic alignment of common hippo genome and cattle genome. The colored syntenic linked lines present the major chromosome fusion events in the common hippo. The densities of coding genes and repeat sequences are calculated with a window size of 5 Mb. I: gene density; II: repeat density; III: CHR-1 density; IV: CHR-2 density; V: CHR-L density; VI: GC content. **b** General overview of genetic innovations in each clade of the Whippomorpha. **c** GO enrichment for the genes nearest to the diverged CNEs of Whippomorpha. **d** The similarity of sequence alignments for the EGF-CNE. H3K4me1 data of human epithelial cells was presented. **e** The mouse phenotype enrichments of cetacean-CNEs. **f** The putative cetacean-CNE concerning the *HAND2* gene. The H3K4me3, H3K27Ac, and CTCF-binding atlas of human cardiac muscle primary cell lines. **g** The sequence alignments for the two divergent CNEs adjacent to *HAND2*. **h** The gene tree shows the evolution of *FGFR2*. **i** The FGFR2 protein sequence alignments. **j** Hi-C map for the common hippo on chr2 from 107.2 Mb to 109.2 Mb with 20 Kb resolution, and the CNE_W156 sequence alignments show that the 49-bp insertion was conserved in cetaceans.

We further identified positively selected genes (PSG), rapidly evolving genes (REG), and conserved non-coding elements (CNE) in Whippomorpha based on the phylogenic tree (Supplementary Fig. S5). Although the inactivation of keratin-related genes occurred independently in cetaceans and hippos^2^, we found that 68 Whippomorpha-CNEs are associated with genes enriched in epidermis and skin development (e.g., *EGF*, *SVEP1*, and *ABCA12*; Fig. 1b–d and Supplementary Fig. S7 and Table S8). One of the most diverged Whippomorpha-CNEs (CNE_WH21) has an insertion of 32-bp nucleotides and locates in the intron of *EGF*, overlapping with a significant H3K4me1 peak of human epithelial cells (Fig. 1d and Supplementary Table S8). As *EGF* is an essential gene for epithelial homeostasis^5^, this insertion might be related to shared skin characters related to aquatic adaptation in Whippomorpha (such as reduced sebaceous glands), pending future experimental studies. Moreover, among 18 PSGs (Supplementary Table S5) and 31 REGs (Supplementary Table S6) identified in the most recent common ancestor (MRCA) of Whippomorpha, *NR3C2* and *SCNN1B* (Supplementary Fig. S8) are essential genes in regulating water and salt balance^6^. These genetic changes imply that the common ancestor of Whippomorpha had already undergone changes in both coding and non-coding regions associated with essential skin functions, whereas further independent skin adaptations to an aquatic habitat occurred by gene inactivation^2^.

We also found adaptively evolved genes associated with innate immunity in the MRCA of Whippomorpha. The most significant PSG is *IFNAR1* (Supplementary Table S5), which has diverse effects on innate immune cells^7^. *IFNAR1*^–/–^ mice are unable to generate intact immune responses^8^. *IFNAR1* is highly conserved, whereas ancestral Whippomorpha had five unique amino acid substitutions (Supplementary Fig. S8a). The N285D mutation significantly alters the structure, causing a β-pleated sheet and an α-helix converted to a loop (Supplementary Fig. S8b) in simulation. Furthermore, genes proximal to Whippomorpha-CNEs enriched in the toll-like receptor signaling pathway (Fig. 1c), which regulates pathogens recognition in the innate immune^9^. Hence, the MRCA of Whippomorpha may have already evolved critical innate immune system adaptations for an aquatic environment.

In contrast with the shared adaptations in the MRCA of Whippomorpha, CNEs unique to hippos and cetaceans show divergent patterns. While the genes nearest to the hippo-CNEs have not significantly enriched GOs (Supplementary Table S7), genes nearest to the cetacean-CNEs enriched in heart and craniofacial morphology (Fig. 1e and Supplementary Table S9). Two cetacean-CNEs are in the upstream (5′ + 465 bp) and downstream (3′ –1426 bp) of the *HAND2* gene, respectively (Fig. 1f, g). The H3K4me3, H3K27AC, and CTCF-binding data of human cardiac muscle primary cells revealed potential regulation effects of both CNEs (Fig. 1f). Another cetacean-CNE (CNE_W78) containing a 21 bp deletion is in the mm87 region (Supplementary Fig. S9), a downstream regulatory element of *HAND2*^10^. *HAND2* is essential for cardiac morphogenesis, especially the formation of ventricular chambers (RV)^11^. These two cetacean-CNEs near *HAND2* might be associated with enlarged RVs and the hearts of most cetaceans. Consistently, we observed PSGs in cetaceans associated with cardiac morphogenesis, such as *NKX2-5* (Supplementary Table S5), a critical component of the cardiac kernel in vertebrates^12^. These divergent CNEs and PSGs reflect a specialized cardiac system in cetaceans at the genetic level.

Both hippos and cetaceans have remarkable and derived craniofacial morphologies to adapt to an aquatic lifestyle. Hippos have dorsally positioned eyes, nostrils and ears, and cetacean nostrils have been re-orientated at the top of the head to make it easier to breathe air while swimming. Therefore, we systematically investigated genes related to craniofacial development. We found that *FGFR2*, which plays an essential role in embryonic development, stands out as a PSG in the MRCA of Whippomorpha, with two positively selected sites, D374E and T394G (Fig. 1h, i). The D374E mutation is in the extracellular juxta-membrane region of *FGFR2*, where two nearby mutations in humans, S372C and Y375C, cause the Beare-Stevenson syndrome entailing abnormal facial features (Supplementary Table S10). The T394G mutation is in the transmembrane region of FGFR2, where three nearby Y381D, G384R, and M391R mutations are associated with craniosynostosis in humans. These results strongly implicate the Whippomorpha specific mutations D374E and T394G in craniofacial rearrangement, providing candidate sites for future functional studies. Another Whippomorpha PSG, *SOX9* (Supplementary Table S5), has also been implicated in early craniofacial development and human craniofacial syndromes^13^. Besides these coding region mutations, we found 7 Whippomorpha-CNEs proximal to genes related to craniofacial morphology (Supplementary Table S8). For example, one CNE (CNE_WH48) has a 12 bp unique deletion in Whippomorpha, located 5981 bp upstream of *FRS2*, and is within a human distal enhancer-like element (EH38E3026373).

Despite some shared craniofacial adaptations among hippos and cetaceans, the fully-aquatic lifestyle of the cetaceans has induced a more substantial craniofacial rearrangement. Interestingly, we found that cetaceans share another conserved amino acid mutation, T268A, in *FGFR2*, located in the Ig-like C2-type 3 domain, which directly interacts with fibroblast growth factors (FGF). Two nearby variants in humans, S267P and T268TG, cause the Crouzon syndrome exhibiting abnormal facial morphology in humans (Supplementary Table S10). Several cetacean-specific rapidly evolving genes (*COLEC11*, *CTSK*, *KAT6A*, *SEC24D*, and *WDR19*; Supplementary Table S6) are also related to craniofacial morphology. Additionally, there are 22 cetacean-CNEs associated with craniofacial development (Supplementary Table S9). For example, one CNE (CNE_W128) exhibits a 10 bp unique deletion in cetaceans, located 42,649 bp upstream of *FGFR2*, and is within a distal enhancer-like element (Supplementary Fig. S10). Another cetacean-CNE (CNE_W156) is in the *VISTA* hs1168 element, correlated with craniofacial development in mouse experimental studies^14^. A craniofacial related gene, *FOXG1*, is located ~420 kb upstream of the CNE_W156, both of which are in the same topologically associating domain (TAD) region of vaquita (Fig. 1j), indicating that *FOXG1* might be the possible regulation target of CNE_W156. For clarity, we summarize the findings regarding Whippomorpha and cetacean-specific coding and non-coding regions implicated in craniofacial development (Supplementary Fig. S11). Together, these results suggest progressive skull evolution in Whippomorpha, with some modifications occurring already in their shared ancestor and others uniquely in the cetacean lineage.

In summary, our results indicate that many genetic changes associated with key aquatic adaptations, including skin, innate immunity, and craniofacial development, were present in the MRCA of Whippomorpha. Moreover, we also identified additional cetacean-specific mutations associated with further aquatic specialization, including an enlarged heart and further specialized craniofacial morphology. Hence, we conclude that many genetic adaptations to aquatic or semi-aquatic habitats occurred in the common ancestor of hippos and cetaceans, and that this animal was already at least semi-aquatic. Our results provide a list of concrete phenotypic adaptations that will be interesting to match with future fossil findings.

## Supplementary information

Supplementary Figures

Supplementary Tables

## Data Availability

The newly assembled genomes and raw data are available in the GenBank (PRJNA780357).
