# Supplementary material for: Progressive evolution of secondary aquatic adaptation in hippos and cetaceans
Source: Cell Discov. 2022 Dec 20;8:134. doi: 10.1038/s41421-022-00483-2 (PMC9768135; doi:10.1038/s41421-022-00483-2)
Supplement: Supplementary file 1 — Supplementary Figures [file 41421_2022_483_MOESM1_ESM.pdf]

This PDF file includes:

Methods

Supplementary Figures S1 to S11.

Additional supplementary information in a single excels:

Supplementary Table S1. Summary of sequencing data

Supplementary Table S2. Summary of genome assemblies

Supplementary Table S3. Summary of repetitive elements in two hippo genomes

Supplementary Table S4. Summary of gene prediction and functional annotation

Supplementary Table S5. Summary of positively selected genes

Supplementary Table S6. Summary of rapidly evolving genes

Supplementary Table S7. Hippos specific CNEs

Supplementary Table S8. Whippomopha specific CNEs

Supplementary Table S9. Whales specific CNEs

Supplementary Table S10. Summary of mutations and phenotype of FGFR2

Supplementary Table S11. Softwares used in this study

Supplementary Table S12. Genomes used in this study

Methods

Genome assembly

Blood samples of common hippo and pygmy hippo were obtained from Guangzhou Zoo separately. Then samples were transferred to NextOmics for DNA extraction and sequencing. Nanopore, HiC, and Illumina libraries were constructed for common hippo samples. Pacbio and BGI libraries were constructed for pygmy hippo samples. Long genome sequencing reads (Pacbio and Nanopore reads) were assembled by NextDenovo (v2.1.0, https://github.com/Nextomics/NextDenovo) with the seed threshold as 14,152bp. The result of nextgraph with the parameter of "-u 1" for further analysis. NextPolish[1] (v2.0.0) was processed to polish the draft genome using the "--best" mode, which uses long and short sequencing reads to polish the genome twice separately. Hi-C reads were mapped to the polished genome by Juicer[2](v1.6). Then we use 3D-DNA[3] (v180419) to scaffold the contigs to 18 chromosomes by inter-contig contacts. Manual validation was processed by Juicebox[4] (v1.13.01). The Hi-C data were re-mapped to the manually calibrated genome, and HiCPlotter[5] (v0.6.6) was used to plot the whole genome heatmap.

The short sequencing reads were mapped to the assembly by BWA mem (v0.7.17) mode with default parameters[6]. The inter-species synteny is also processed to verify the genome quality. Both genomes are aligned to the cattle genome (GCA_002263795.2) by lastz (v1.04.03) with the parameter "O=400 E=30 M=254". Alignment blocks are chained by axtChain with default parameter, and then chains are netted into whole genome alignments by chainNet with default parameters. Genomic synteny was visualized by Circos[7]. Previous inter-species chromosome FISH results between pygmy hippo and cattle were used for manual check. BUSCO[8] (v3.0.2) analysis was possessed at genome mode to assess the genome completeness with the "ODB9_mammalia" dataset.

Genome annotation

Repeat elements in the genome were found by RepeatMasker[9] (v4.1.2) with RepBase, RepeatModeller[10] (v2.0.2a), TRF[11] (v4.09), LTR finder[12] (v1.07), and RepeatProteinMasker[9] (v4.1.2), separately. Then BEDTools[13] (v2.30.0) was used to merge the result and mask the repetitive region of the genome. Several methods were combined to predict the protein coding gene in the genome. Firstly, the RNA-seq data

from NCBI (SRR8270566) was mapped to the genome with HISAT2[14]. Stringite2[15] was used to assemble the alignment into transcripts. CDS in transcripts were predicted by TransDecoder (v5.5.0, https://github.com/TransDecoder/TransDecoder). Later, the protein sequences from ensemble annotation of the mouse, human, and cattle were mapped to the genome by tBLASTn[16] with an E value of 1e-5. Then we use Genewise[17] to predict gene models based on protein alignment. Thirdly, the genome was aligned to each genome of human, cattle, vaquita, and sperm whale by our WGA pipeline. Gene projection was inferred by TOGA[18] (v1.0.1) with the target genome annotation from ENSEMBL. Besides, *de novo* gene prediction was made by AUGUSTUS[19] (v3.3.3) with the default parameter. At last, we used EvidenceModeller[20] to combine all the gene models. We use BUSCO[8] (v3.0.2) with the "ODB9_mammalia" dataset to validate the completeness of our annotation gene set. Functional annotation was processed by Entap[21] (v0.10.8) at "-runP" mode with the default parameter. The Refseq protein database, NR database, and Uniprot Swissprot database are used as the similarity annotation database.

Pairwise whole genome alignments

All whole genome alignments in this work were done with the UCSC whole genome alignment pipeline: 1) Soft masked query and target genomes were aligned by lastz (v1.04.03) with parameter "O=400 E=30 M=254"; 2) Alignment blocks were chained into chains by axtChain with paremeter "-linearGap=loose"; 3) chainNet and netSyntenic were processed to netting chains to syntenic whole genome alignment.

Historical population dynamic inferences

50 × small sequencing reads were mapped to the genome by Bowtie2[22] (v2.4.4). Samtools[23] (v1.14) was used to sort and index the alignment BAM files. Then genotype likelihoods of sites with mapping quality greater than 50 were estimated by Samtools mpileup command. BCFtools[24] (v1.10.1) with default parameters was used to call SNPs. We used vcfutil.pl[25] to generate the diploid genome sequence, with parameters: "-d 10 -D 100" to exclude the sites with root-mean-square mapping quality < 10 and mapping depths > 100 or <10. The format of the consensus sequence was transformed to PSMC input format by fq2psmcfa with parameters: -q20. After the transformation, the population size histories were inferred by PSMC[26] (v0.6.5) with the parameters: "psmc -N25 -t15 -r5 -p 4+25*2+4+6 ", and these results were then scaled to absolute time and effective population sizes using generation times and

estimated mutation rates (per generation) by running "psmc2history.pl" (with the default parameters) and "history2ms.pl" (with the parameters: -g estimates_generation_time -u neutral_mutation_rates).

Identification of orthologous genes

Each genome of dog, camel, pig, sperm whale, vaquita, common hippo, and pygmy hippo genome (Table S12) was aligned to the cattle genome by the whole genome alignment pipeline mentioned before. Then TOGA[18] (v1.0.1) was used to identify the orthologs of cattle's genes (ensemble bos10) with default parameters. Result with the label "one 2 one" and "complete" was used as the orthologs of the query species for downstream analysis.

Phylogenic tree construction.

CDS sequences of each 11,274 orthologous genes are aligned by PRANK[27] (v170427) with parameters: "-f=fasta -codon +F". The four-fold degenerate (4d) sites were extracted. A total of 1,715,877 sites were concatenated and used for constructing an ML phylogenetic tree by RAxML-ng[28] (with parameters: "--model GTR+G --bs-trees 200"). 200 bootstrap replicates were run to calculate the support for each node.

Divergence time calibration

MCMCTREE in PAML package[29] (v4.9) was used with five nodes calibrated by fossil data from TimeTree website (https://timetree.org) to estimate the divergence time of nodes in the phylogeny tree based on 4d sites.

Identification of positively selected and rapidly evolving genes.

The ortholog genes are used to identify positively selected genes (PSGs) and rapidly evolving genes (REGs). To identify the PSGs, we firstly estimated the lineage-specific evolutionary rates for each ortholog using the Codeml module with a free-ratio model (model=1) in the PAML software package[29] (v4.9). Then we used the branch-site model (model=2) to identify genes having a higher ω (*d*N/*d*S) than the rest of the lineage in the tree. In the branch-site model, we used five different foreground species sets (1: hippo,2: hippo + pygmy hippo,3: pygmy hippo, 4: sperm whale + vaquita, 5: hippo + pygmy hippo + sperm whale + vaquita) to identify PSGs of different nodes. A likelihood ratio test (LRT) was conducted to compare the above two models, and the genes with a *p*-value (chi-square statistics) less than 0.01 were regarded as PSGs. To identify the rapidly evolving genes (REGs), we tested the same

orthologous gene set using a branch model with the null model (model=0), assuming that all branches have evolved at the same rate and an alternative model which allows the foreground branch evolves at a different rate (model=2). Similarly, the LRT was used to compare the two models, and the genes with *p* value less than 0.01 were treated as REGs. We then performed the KEGG pathway, Reactome pathway, and Gene Ontology (GO) enrichment analysis using a web-based tool Metascape[30] with parameters of min Overlap: 3, *p* value cutoff: 0.01, min enrichment: 1.5.

Identification of species-specific mutation sites.

We used in-house scripts to identify the species-specific mutation sites in orthologous genes. Five types of ingroup sets (1: hippo,2: hippo, pygmy hippo,3: pygmy hippo, 4: sperm whale, vaquita, 5: common hippo, pygmy hippo, sperm whale, vaquita) were used. We go through every site in the alignment of the orthologous genes and identify species-specific mutation sites with the following criteria: 1) Site is identity in the ingroup and outgroup separately, which means that over 80% of species in the group share the identity site; 2) Site is located at the pfam domain; 3) Site is totally different between ingroup and outgroup species. We verified the diverged sites in additional genomes of chicken, lizard, elephant, opossum, and narwhal (Table S12).

Identification of topological associated domain (TAD).

The HiC-Pro pipeline[31] (v3.0.0, with default parameter) was used to construct the contact matrices based on the high-quality Hi-C reads and chromosome-level genomes. This pipeline first mapped the Hi-C reads of each sample to its reference genome using Bowtie2[22] (v2.4.4) software. Then the unmapped reads, singletons, and multi-hits reads were filtered out. The remaining reads were assigned to the restriction fragments (restriction enzyme: MboI), and the valid reads pairs were obtained by filtering out the dangling end, self-circle, ligation, dumped pairs, and PCR artifacts. These valid read pairs were used to construct the raw contact matrices at different resolutions (1 Mb, 100 kb, 40 kb, and 20kb). The raw contact matrices were then normalized using the iterative correction and eigenvector decomposition (ICE) method. We detected TAD boundaries based on the normalized contact matrix at 40 kb resolution using the insulation score method with parameter (--is 1000000 –ids 240000). Hi-C heatmap of the TAD region was generated by HiCPlotter[5] (v0.6.6) at the resolution of 20 kb with a Hi-C matrix generated from Hi-C Pro.

Identification of CNEs

The genomes of human, mouse, dog, horse, camel, pig, cow, goat, common hippo, pygmy hippo, blue whale, sperm whale, beluga whale, and vaquita were used to identify the CNEs (Table S12). All other genomes were aligned against the human genome (hg38) separately with the pre-mentioned WGA methods. Then all maf-format alignments were merged by MULTIZ[32] (2009-Jan-21) using the human genome as the reference. After that, a series of subroutines of PHAST[33] (v1.4) (phyloFit, phyloBoot, phastCons) (with parameters -estimate-rho -no-post-probs -most-conserved -score) was used to estimate and filter conserved regions of the 14 species. ANNOVAR[34] (v2019) was used to split coding and non-coding regions according to the coordinates in the GFF file from human hg38 and retains non-coding regions for subsequent analysis of conserved elements. Sequences with similarity higher than 80% in at least 7 of 8 terrestrial mammals are retained for subsequent analysis, and the corresponding conserved elements in these terrestrial mammals are present in four aquatic mammals with contiguous mutations >= 10 bp (including insertions or deletions or mutations) to be remained for the potential highly conserved non-coding elements in Whippomorpha. Based on the positions of genes in the GFF files in these species to determine whether these potential CNEs are in the vicinity of the same gene, retaining the CNEs in the vicinity of the same gene in at least 10 species as potential regulatory elements for that gene. The functional enrichment analysis was conducted in GREAT[35] (v4.0.4), and duplicated items are curated.

IFNAR1 protein structure modeling.

The structure of IFNAR1 is constructed by homolog modeling using Phyre2[36] (v2.0). The human IFNAR1 was used as the template structure for modeling. The result with the maximal score was selected as the best structure and visualized by UCSF Chimera.

Epigenomic data visualization.

The epigenomic raw data were downloaded from ENCODE database. All epigenomic profile figure in this study was generated in WashU Epigenome Browser (http://epigenomegateway.wustl.edu/browser/).

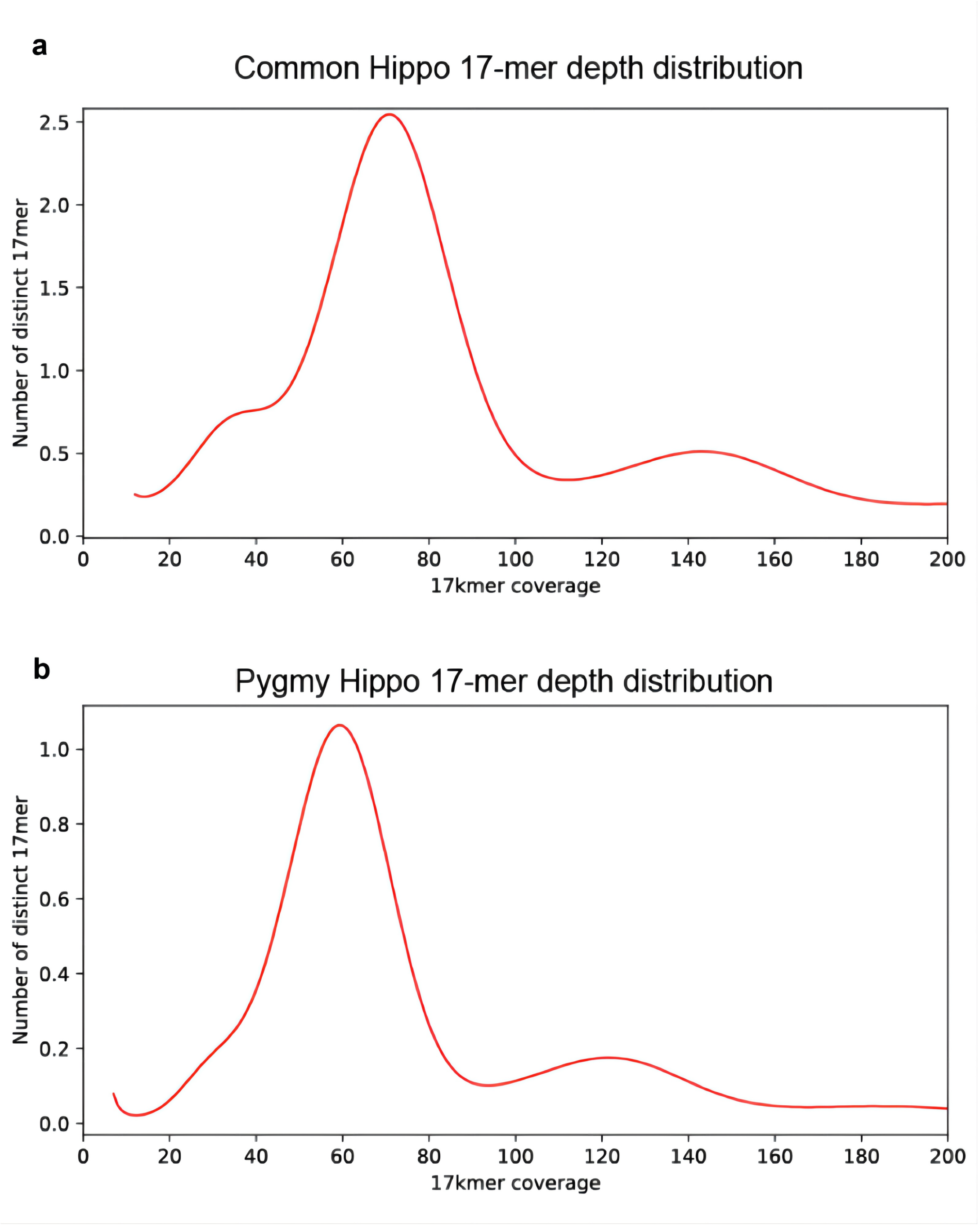

Supplementary Figure S1. *K*-mer depth distribution profiles of common hippo(**a**) and pygmy hippo (**b**). The estimated genome sizes are2.51 Gb and 2.48 Gb for common hippo and pygmy hippo, respectively.

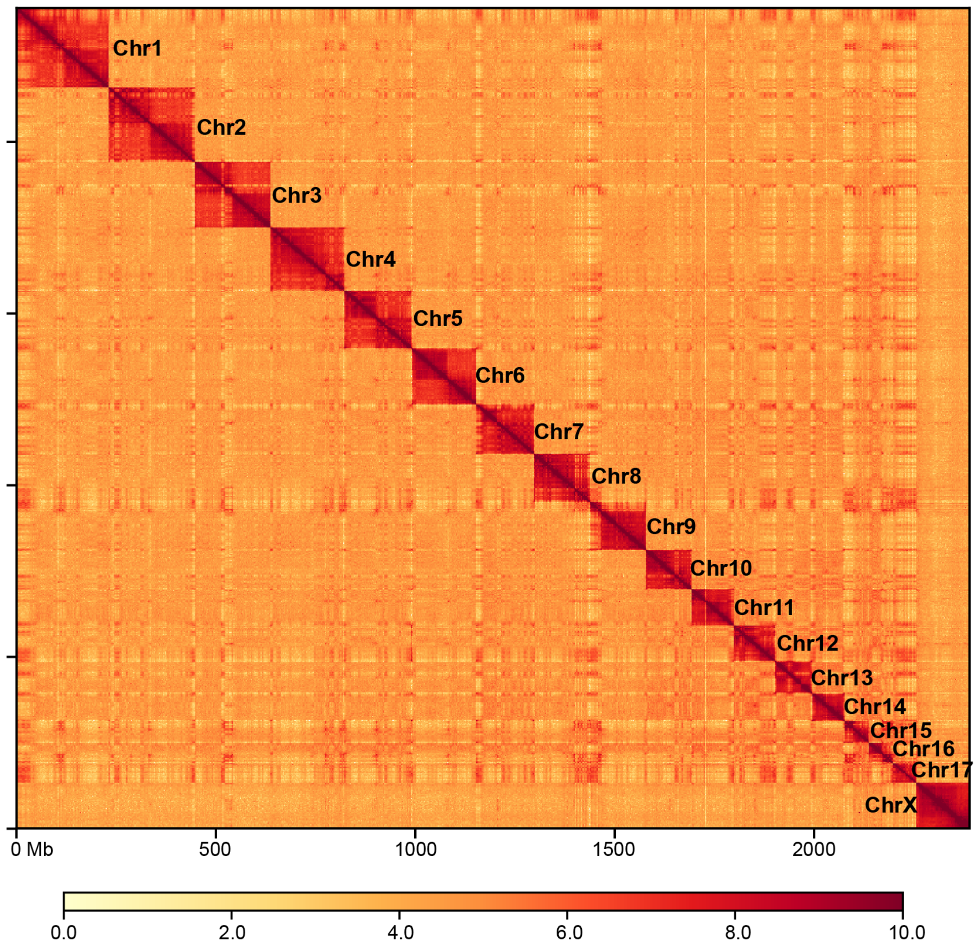

Supplementary Figure S2. Hi-C heatmap of common hippo genome assembly is plotted using HiCPlotter. The chromosomes are ordered by size. ChrX was identified according to the whole genome alignments with the cattle genome (Figure 1a).

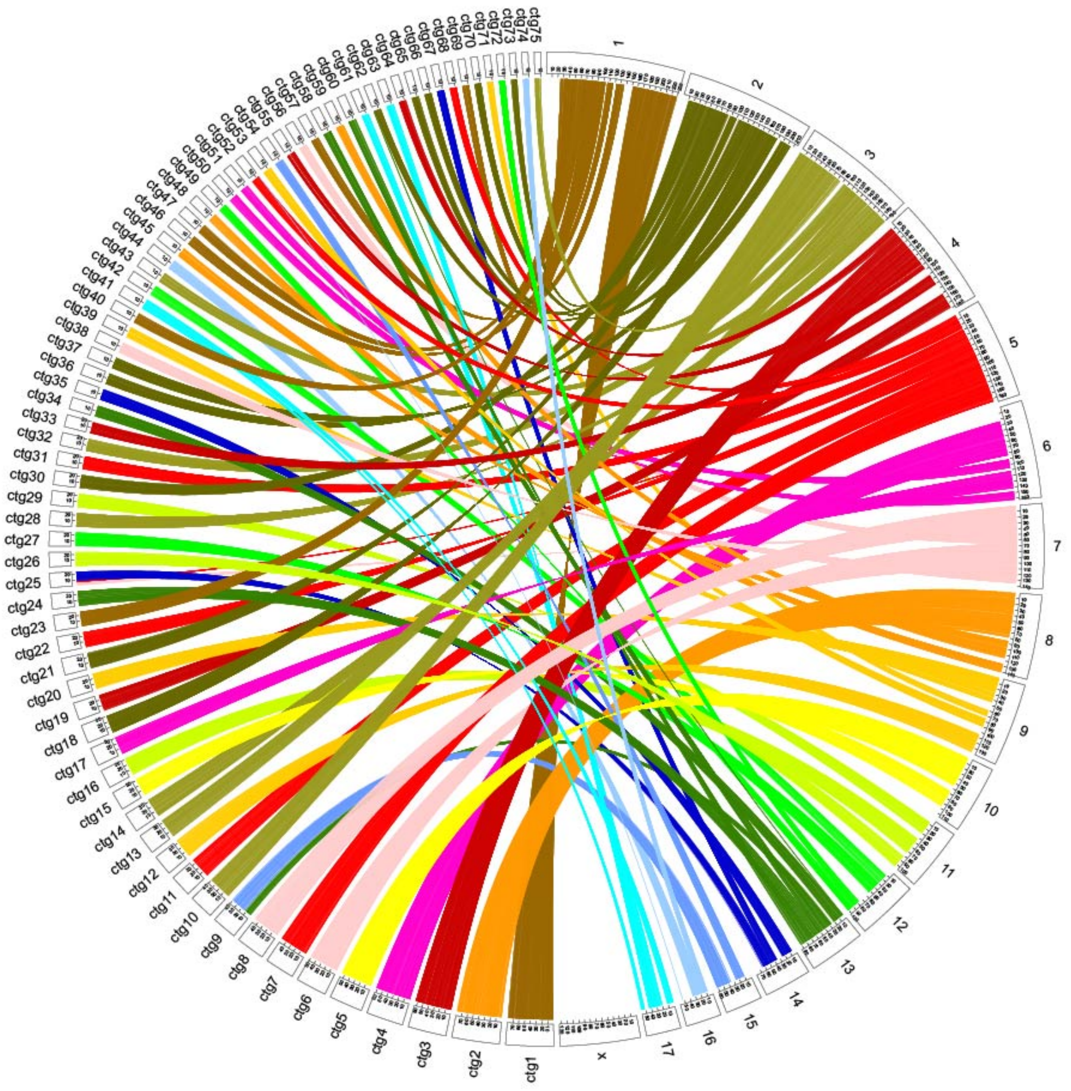

Supplementary Figure S3. Circos plot shows the synteny between contigs of pygmy hippo and anchored pseudo-chromosomes. The contigs of pygmy hippo are labeled on the left, and the pseudo-chromosomes are on the right.

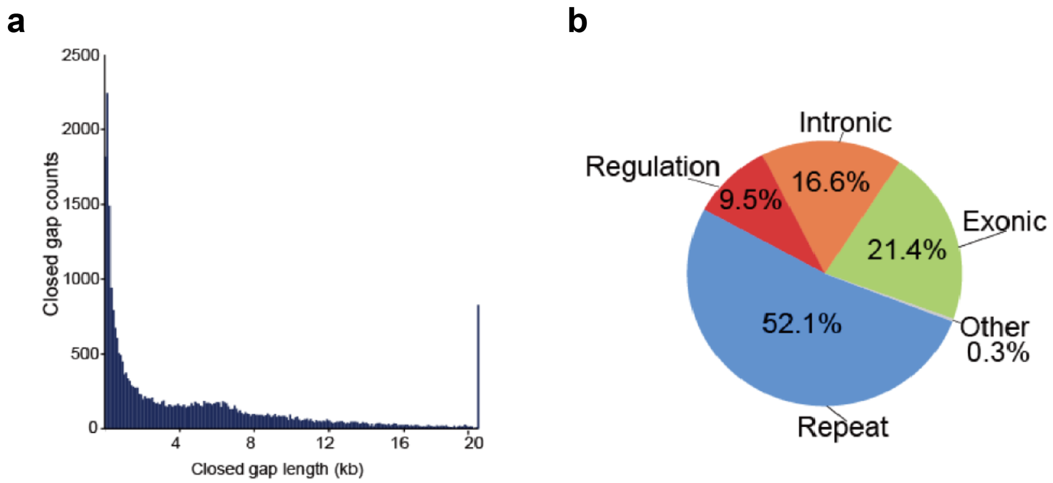

Supplementary Figure S4. The figure presents length distribution (**a**) and location distribution (**b**) of closed gaps in common hippo genomes compared to the GCA_004027065.1 assembly.

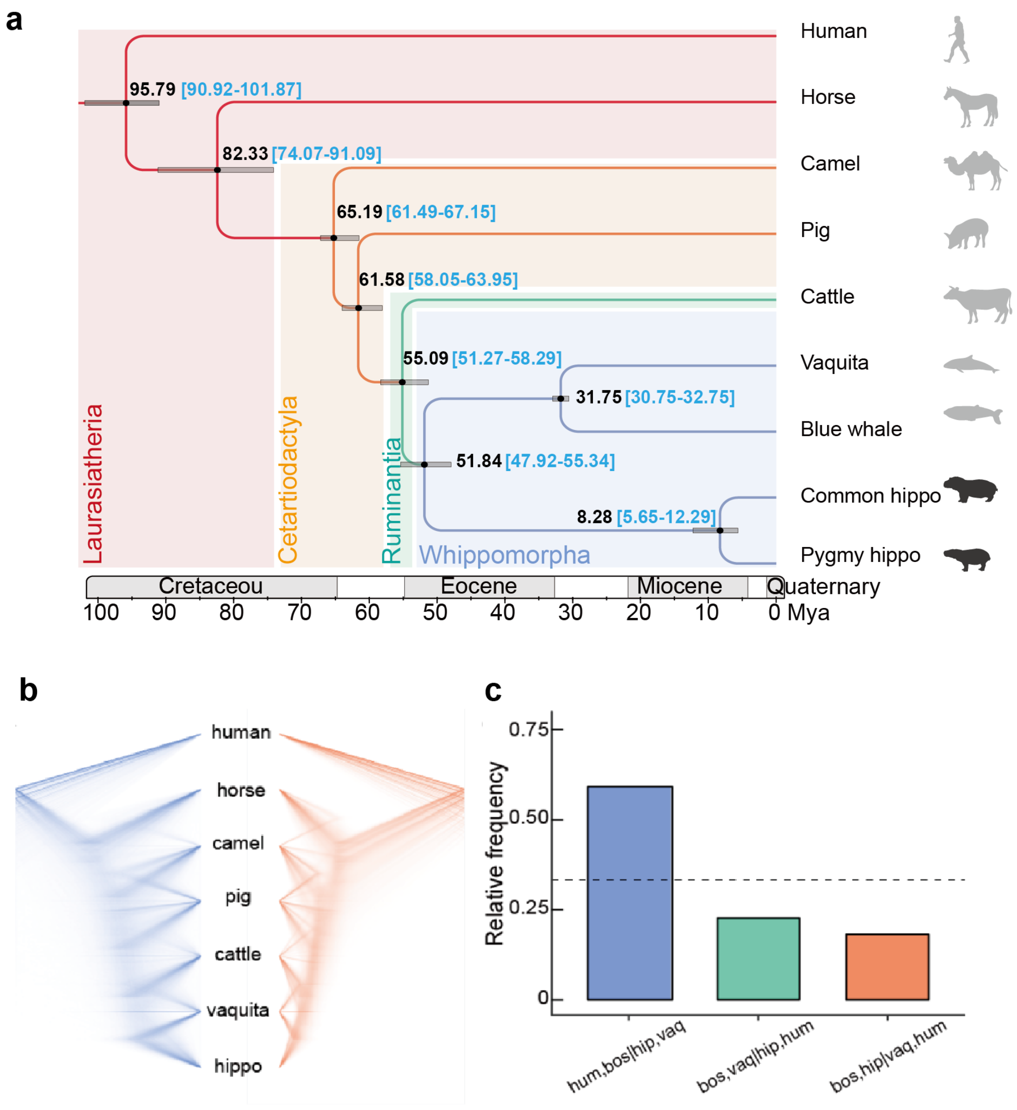

Supplementary Figure S5. (**a**) Phylogenetic relationships of the hippos and whales. The divergence time was indicated by the black number near each node, while the 95% CI was indicated by the blue rectangle, and its specific value was colored in red under the corresponding divergence time. Four softbound calibration time points were applied: pygmy hippo and common hippo (5.7 - 12.7 Mya), vaquita and blue whale (31.1 - 35.0 Mya), human and horse (91 - 102 Mya), camel and pig (61 - 67 Mya). (**b**) Superimposed ultrametric gene trees in a consensus DensiTree plot. The observed gene trees and multispecies coalescent simulated gene trees are shown as blue and orange, respectively. (**c**) Typology frequencies among cattle, hippo, and vaquita of all gene trees are presented.

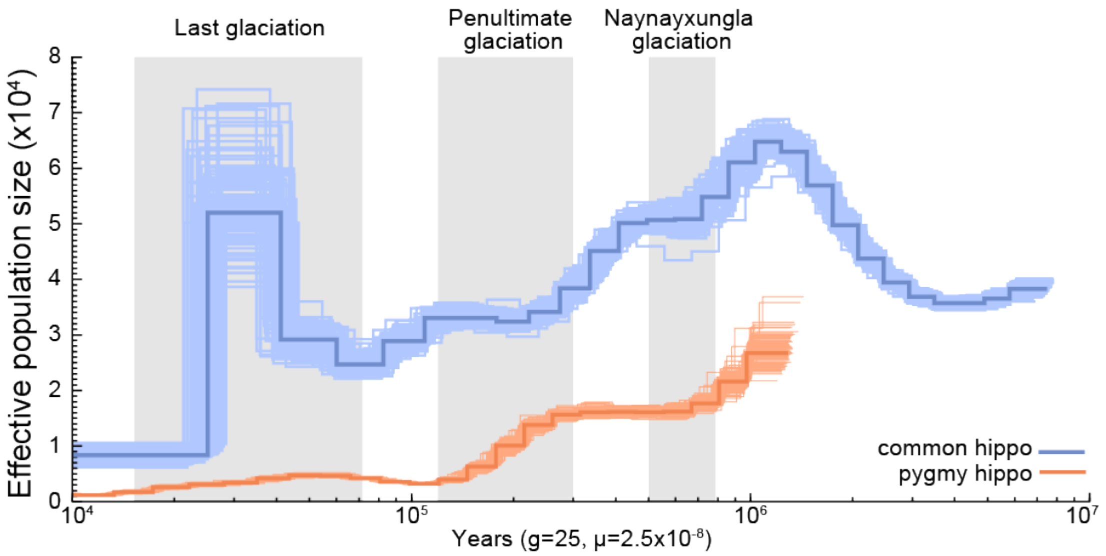

Supplementary Figure S6. Demographic histories for common hippo and pygmy hippo are inferred by PSMC based on Illumina data.

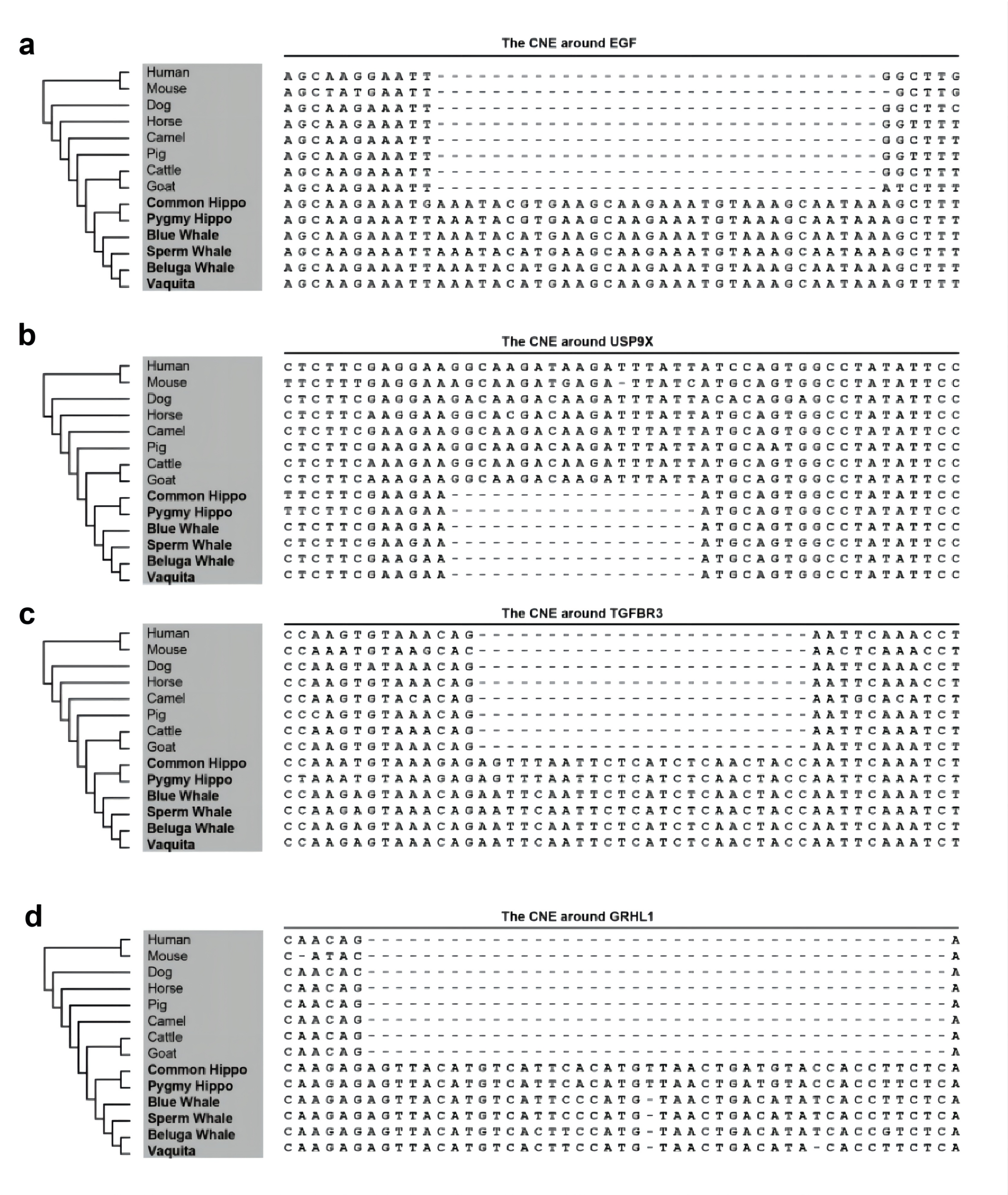

Supplementary Figure S7. Multiple sequence alignments of Whippophoma specific CNEs around *EGF* (**a**), *USP9X* (**b**), *TGFBR3* (**c**), and *GRHL1* (**d**) genes are presented.

a

	NR3C2					SCNN1B			IFNAR1				
	45	157	158	384	787	89	100	604	66	262	285	293	304
Chicken	I	Q	N	I	L	T	A	-	Q	L	N	L	Y
Lizard	I	Q	N	I	L	T	A	R	Q	L	H	L	Y
Opossum	I	Q	N	I	L	T	A	Y	Q	L	N	F	Y
Elephant	I	Q	N	I	L	T	A	Y	Q	L	N	F	Y
Human	I	Q	N	I	L	T	A	Y	Q	L	N	F	Y
Dog	I	Q	N	I	L	T	A	Y	Q	L	N	F	Y
Horse	I	Q	N	I	I	T	A	Y	Q	L	N	F	Y
Camel	I	Q	N	I	L	T	A	Y	Q	L	N	F	Y
Pig	I	Q	N	I	L	T	A	Y	Q	L	N	F	F
Cattle	I	Q	N	I	L	T	A	Y	Q	L	N	F	Y
Goat	I	Q	N	I	L	T	A	Y	Q	L	N	F	Y
Common Hippo	V	P	H	V	I	A	T	H	K	S	D	I	H
Pygmy Hippo	V	P	H	V	I	A	T	H	K	S	D	I	H
Blue Whale	V	P	H	V	I	A	T	H	K	S	D	I	H
Sperm Whale	V	P	H	V	I	A	T	H	K	S	D	I	H
Beluga Whale	V	P	H	V	I	A	T	H	K	S	D	I	H
Vaquita	V	P	H	V	I	A	T	H	K	S	D	I	H
Narwhal	V	P	H	V	I	A	T	H	K	S	D	I	H

b

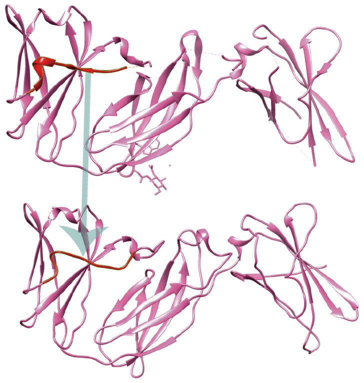

Supplementary Figure S8. (a) Sequence alignments of the three most significantly positively selected genes. **(b)** 3D structure of human IFNAR1 protein and structure simulated by a homologous approach for human IFNAR1 protein with the N285D mutation.

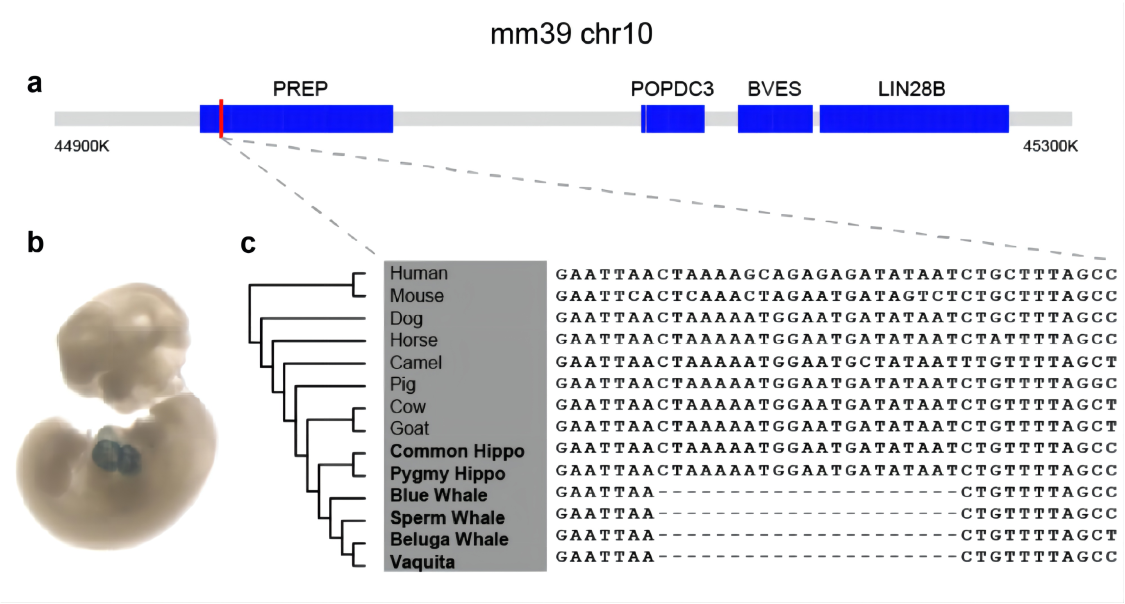

Supplementary Figure S9. A whales-specific CNE (CNE_W78) is located in mm87 enhancer region. (**a**) The mm87 enchancer locates in the intron of *PREP* gene in the mouse genome (version: mm39). (**b**) VISTA experiment result showed the probable regulation effects of heart development for mm87. (**c**) Multiple sequence alignments show the whales specific CNE harbor an indel of 21 nucleotides in mm87 region.

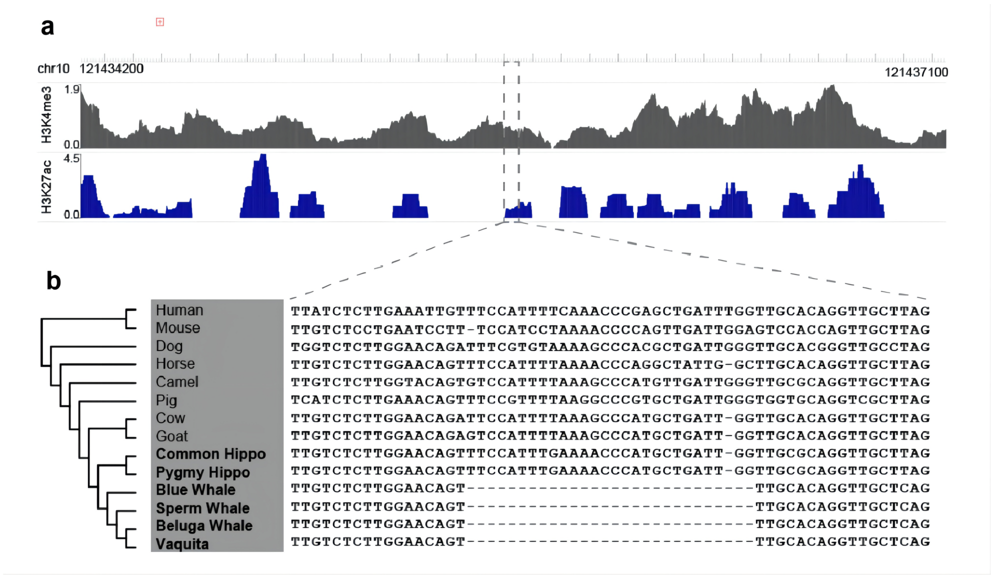

Supplementary Figure S10. The whales-specific CNE (CNE_W128) located at the upstream (5' +42,649 bp) of the *FGFR2* gene. (**a**) The genomic location and epigenomic signals (NCFF753DQP and ENCFF664DOZ) in the mesendoderm cell of this CNE are presented. This region is predicted as a distal enhancer in the human ENCODE database. (**b**) Multiple sequence alignments of this CNE showed the specific indel of 28 nucleotides in whales.

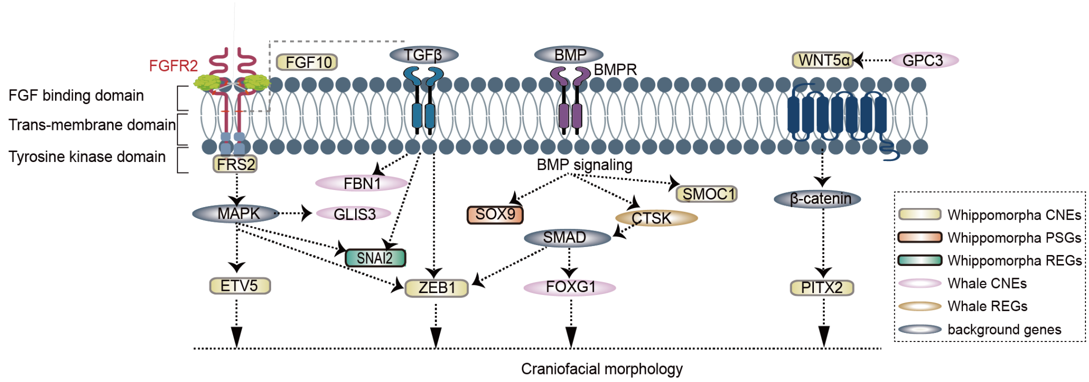

Supplementary Figure S11. The pathway of the most divergent genes and CNEs in the Whippomorpha and Cetacean lineage, concerning with the gene networks of craniofacial development.

References.

1 Hu, J., Fan, J., Sun, Z. & Liu, S. NextPolish: a fast and efficient genome polishing tool for long-read assembly. *Bioinformatics* **36**, 2253-2255 (2020).

2 Durand, N. C., Shamim, M. S., Machol, I., Rao, S. S., Huntley, M. H. *et al.* Juicer Provides a One-Click System for Analyzing Loop-Resolution Hi-C Experiments. *Cell Syst* **3**, 95-98 (2016).

3 Dudchenko, O., Batra, S. S., Omer, A. D., Nyquist, S. K., Hoeger, M. *et al.* De novo assembly of the Aedes aegypti genome using Hi-C yields chromosome-length scaffolds. *Science* **356**, 92-95 (2017).

4 Durand, N. C., Robinson, J. T., Shamim, M. S., Machol, I., Mesirov, J. P. *et al.* Juicebox Provides a Visualization System for Hi-C Contact Maps with Unlimited Zoom. *Cell Syst* **3**, 99-101 (2016).

5 Akdemir, K. C. & Chin, L. HiCPlotter integrates genomic data with interaction matrices. *Genome Biol* **16**, 198 (2015).

6 Li, H. & Durbin, R. Fast and accurate short read alignment with Burrows-Wheeler transform. *Bioinformatics* **25**, 1754-1760 (2009).

7 Krzywinski, M., Schein, J., Birol, I., Connors, J., Gascoyne, R. *et al.* Circos: an information aesthetic for comparative genomics. *Genome Res* **19**, 1639-1645 (2009).

8 Seppey, M., Manni, M. & Zdobnov, E. M. BUSCO: Assessing Genome Assembly and Annotation Completeness. *Methods Mol Biol* **1962**, 227-245 (2019).

9 Tarailo-Graovac, M. & Chen, N. Using RepeatMasker to identify repetitive elements in genomic sequences. *Curr Protoc Bioinformatics* **Chapter 4**, Unit 4 10 (2009).

10 Flynn, J. M., Hubley, R., Goubert, C., Rosen, J., Clark, A. G. *et al.* RepeatModeler2 for automated genomic discovery of transposable element families. *Proceedings of the National Academy of Sciences of the United States of America* **117**, 9451-9457 (2020).

11 Benson, G. Tandem repeats finder: a program to analyze DNA sequences. *Nucleic Acids Res* **27**, 573-580 (1999).

12 Xu, Z. & Wang, H. LTR_FINDER: an efficient tool for the prediction of full-length LTR retrotransposons. *Nucleic Acids Res* **35**, W265-268 (2007).

13 Quinlan, A. R. BEDTools: The Swiss-Army Tool for Genome Feature Analysis. *Curr Protoc Bioinformatics* **47**, 11 12 11-34 (2014).

14 Kim, D., Paggi, J. M., Park, C., Bennett, C. & Salzberg, S. L. Graph-based genome alignment and genotyping with HISAT2 and HISAT-genotype. *Nat Biotechnol* **37**, 907-915 (2019).

15 Kovaka, S., Zimin, A. V., Pertea, G. M., Razaghi, R., Salzberg, S. L. *et al.* Transcriptome assembly from long-read RNA-seq alignments with StringTie2. *Genome Biol* **20**, 278 (2019).

16 Camacho, C., Coulouris, G., Avagyan, V., Ma, N., Papadopoulos, J. *et al.* BLAST+: architecture and applications. *BMC Bioinformatics* **10**, 421 (2009).

17 Birney, E., Clamp, M. & Durbin, R. GeneWise and Genomewise. *Genome Res* **14**, 988-995 (2004).

18 Jebb, D., Huang, Z., Pippel, M., Hughes, G. M., Lavrichenko, K. *et al.* Six reference-quality genomes reveal evolution of bat adaptations. *Nature* **583**, 578-584 (2020).

19 Stanke, M., Keller, O., Gunduz, I., Hayes, A., Waack, S. *et al.* AUGUSTUS: ab initio prediction of alternative transcripts. *Nucleic Acids Res* **34**, W435-439 (2006).

20 Haas, B. J., Salzberg, S. L., Zhu, W., Pertea, M., Allen, J. E. *et al.* Automated eukaryotic gene structure annotation using EVidenceModeler and the Program to Assemble Spliced Alignments. *Genome Biol* **9**, R7 (2008).

21 Hart, A. J., Ginzburg, S., Xu, M. S., Fisher, C. R., Rahmatpour, N. *et al.* EnTAP: Bringing faster and smarter functional annotation to non-model eukaryotic transcriptomes. *Mol Ecol Resour* **20**, 591-604 (2020).

22 Langmead, B. & Salzberg, S. L. Fast gapped-read alignment with Bowtie 2. *Nat Methods* **9**, 357-359 (2012).

23 Li, H., Handsaker, B., Wysoker, A., Fennell, T., Ruan, J. *et al.* The Sequence Alignment/Map format and SAMtools. *Bioinformatics* **25**, 2078-2079 (2009).

24 Li, H. A statistical framework for SNP calling, mutation discovery, association mapping and population genetical parameter estimation from sequencing data. *Bioinformatics* **27**, 2987-2993 (2011).

25 Hickey, G., Heller, D., Monlong, J., Sibbesen, J. A., Siren, J. *et al.* Genotyping structural variants in pangenome graphs using the vg toolkit. *Genome Biol* **21**, 35 (2020).

26 Li, H. & Durbin, R. Inference of human population history from individual whole-genome sequences. *Nature* **475**, 493-496 (2011).

27 Loytynoja, A. Phylogeny-Aware Alignment with PRANK and PAGAN. *Methods Mol Biol* **2231**, 17-37 (2021).

28 Kozlov, A. M., Darriba, D., Flouri, T., Morel, B. & Stamatakis, A. RAxML-NG: a fast, scalable and user-friendly tool for maximum likelihood phylogenetic inference. *Bioinformatics* **35**, 4453-4455 (2019).

29 Yang, Z. PAML 4: phylogenetic analysis by maximum likelihood. *Molecular biology and evolution* **24**, 1586-1591 (2007).

30 Zhou, Y., Zhou, B., Pache, L., Chang, M., Khodabakhshi, A. H. *et al.* Metascape provides a biologist-oriented resource for the analysis of systems-level datasets. *Nat Commun* **10**, 1523 (2019).

31 Servant, N., Varoquaux, N., Lajoie, B. R., Viara, E., Chen, C. J. *et al.* HiC-Pro: an optimized and flexible pipeline for Hi-C data processing. *Genome Biol* **16**, 259 (2015).

32 Blanchette, M., Kent, W. J., Riemer, C., Elnitski, L., Smit, A. F. *et al.* Aligning multiple genomic sequences with the threaded blockset aligner. *Genome Res* **14**, 708-715 (2004).

33 Hubisz, M. J., Pollard, K. S. & Siepel, A. PHAST and RPHAST: phylogenetic analysis with space/time models. *Brief Bioinform* **12**, 41-51 (2011).

34 Wang, K., Li, M. & Hakonarson, H. ANNOVAR: functional annotation of genetic variants from high-throughput sequencing data. *Nucleic Acids Res* **38**, e164 (2010).

35 McLean, C. Y., Bristor, D., Hiller, M., Clarke, S. L., Schaar, B. T. *et al.* GREAT improves functional interpretation of cis-regulatory regions. *Nat Biotechnol* **28**, 495-501 (2010).

36 Kelley, L. A., Mezulis, S., Yates, C. M., Wass, M. N. & Sternberg, M. J. The Phyre2 web portal for protein modeling, prediction and analysis. *Nat Protoc* **10**, 845-858 (2015).
